# Two randomised phase II trials of subcutaneous interleukin-2 and histamine dihydrochloride in patients with metastatic renal cell carcinoma

**DOI:** 10.1038/sj.bjc.6602768

**Published:** 2005-08-30

**Authors:** F Donskov, M Middleton, K Fode, P Meldgaard, W Mansoor, J Lawrance, N Thatcher, H Nellemann, H von der Maase

**Affiliations:** 1 Department of Oncology, Aarhus University Hospital, Nørrebrogade 44, 8000 Aarhus C, Denmark; 2 Department of Medical Oncology, Christie Hospital, Manchester, UK; 3 Department of Radiology, Aarhus University Hospital, Aarhus, Denmark; 4 Department of Radiology, Christie Hospital, Manchester, UK

**Keywords:** histamine dihydrochloride, interleukin-2, renal cell carcinoma, randomised, oxidative stress

## Abstract

Histamine inhibits formation and release of phagocyte-derived reactive oxygen species, and thereby protects natural killer and T cells against oxidative damage. Thus, the addition of histamine may potentially improve the efficacy of interleukin-2 (IL-2). Two randomised phase II trials of IL-2 with or without histamine dihydrochloride (HDC) in patients with metastatic renal cell carcinoma (mRCC) were run in parallel. A total of 41 patients were included in Manchester, UK and 63 in Aarhus, Denmark. The self-administered, outpatient regimen included IL-2 as a fixed dose, 18 MIU s.c. once daily, 5 days per week for 3 weeks followed by 2 weeks rest. Histamine dihydrochloride was added twice daily, 1.0 mg s.c., concomitantly with IL-2. A maximum of four cycles were given. The Danish study showed a statistically significant 1-year survival benefit (76 *vs* 47%, *P*=0.03), a trend towards benefit in both median survival (18.3 *vs* 11.4 months, *P*=0.07), time to PD (4.5 *vs* 2.2 months, *P*=0.13) and clinical benefit (CR+PR+SD) (58 *vs* 37%, *P*=0.10) in favour of IL-2/HDC, whereas the UK study was negative for all end points. Only three patients had grade 4 toxicity; however, two were fatal. A randomised phase III trial is warranted to clarify the potential role of adding histamine to IL-2 in mRCC.

Patients with untreated metastatic renal cell carcinoma (mRCC) have a poor prognosis with a median survival of about 8 months (Haas *et al*, 1993), and only 10% survive beyond 3 years (Elson *et al*, 1988). The results remain poor because mRCC is highly refractory to therapy, including radiation, hormones and chemotherapy (Motzer and Russo, 2000). However, immunological manipulation using interleukin-2 (IL-2) mediate durable tumour regression in 5–10% of patients lasting 5 and 10 years, and this subgroup of patients are probably cured (Bordin *et al*, 2000; Fisher *et al*, 2000; Negrier *et al*, 2000; Atzpodien *et al*, 2002). Despite this important improvement, the vast majority of patients will die of their disease and therefore novel treatment strategies or identification of new agents with better antitumour activity remain a high priority.

The explanation for the low level of treatment success following IL-2 based immunotherapy is probably extremely complex. However, the new paradigm of tumours arising and persisting in the setting of chronic inflammation (Pollard, 2004; Vakkila and Lotze, 2004) may represent one of several factors of importance for treatment failure. Among macrophage and neutrophil products, reactive oxygen species (ROS) may not only induce genomic instability (O'Byrne and Dalgleish, 2001) but also damage antitumour immune effector cells, especially natural killer (NK) and T cells (Seaman *et al*, 1982; Hellstrand *et al*, 1994a; Hansson *et al*, 1996a). Intratumoural macrophages isolated from melanoma metastases inhibit NK cell function by the release of ROS (Kono *et al*, 1996). Intratumoral NK and T cells isolated from mRCC show sign of oxidative damage (Finke *et al*, 1993; Tartour *et al*, 1995). Interleukin-2 cannot activate NK cells *in vitr*o in the presence of monocytes or macrophages (Hellstrand and Hermodsson, 1990). However, histamine dihydrochloride (HDC), acting via H_2_-type receptors on phagocytes, has been shown to inhibit the ROS-production by phagocytes, and therefore protects NK and T cells from inhibition and apoptosis. Reversal of this oxidative suppression of NK and T cells may, thus, be important to enhance the immune response with IL-2 (Hellstrand *et al*, 2000; Hellstrand, 2002). We have explored this potential mechanism clinically in the present two randomised phase II trials of IL-2 with or without HDC in mRCC.

## PATIENTS AND METHODS

### Aims and objectives

Two parallel randomised, prospective, open-label, phase II trials were conducted, one in Manchester, UK and one in Aarhus, Denmark to compare IL-2/HDC *vs* IL-2-alone as first-line treatment for mRCC. The two studies had a similar study design and patient selection criteria. Primary objectives were response and toxicity. Secondary objectives were time to disease progression, overall survival and 1-year survival.

### Patients

The trials were approved by the local ethics committees and the National Medical Agencies. All subjects gave written informed consent before inclusion. Main inclusion criteria were inoperable, bidimensionally measurable, histologically confirmed mRCC, 18–75 years of age, Karnofsky performance status ⩾70, life expectancy >3 months; haemoglobin >10.0 g dl^−1^; white blood cell count >3.0 × 10^9^ cells l^−1^; platelet count >100 × 10^9^ l^−1^; partial thromboplastin time and creatinine <1.5 times the upper limit of normal; serum bilirubin <1.25 the upper limit of normal. Main exclusion criteria were brain metastasis, central nervous system disorders, psychiatric disability, pheochromocytoma, glaucoma, abnormal cardiac function, asthma or systemic allergic reaction treated within the last 5 years, bleeding ulcer disease, infections requiring antibiotics, prior chemotherapy, immunotherapy or extensive radiotherapy in the last 4 weeks and ongoing active malignancies except *in situ* carcinoma of the cervix or localised carcinomas of the skin.

Beta-blocker medications, H_2_ receptor antagonists and steroidal medications were not allowed. H_1_ receptor antagonists were allowed <5 days to treat skin itching.

### Treatment

Patients were consecutively randomised by center to receive either IL-2/HDC or IL-2 alone. One cycle consisted of IL-2 (Aldesleukin, rIL-2, Proleukin®, Chiron, The Netherlands) as a fixed dose, 18 MIU s.c. once daily, 5 days per week for 3 weeks followed by 2 weeks rest. HDC (Ceplene™, supplied by Maxim Pharmaceuticals Inc, San Diego, USA) 1.0 mg, was added twice daily by a slow 20-min injection s.c., concomitantly with IL-2. Patients were evaluated for objective response every two cycles. A maximum of four treatment cycles was given.

Due to the outpatient nature of this protocol, patients received instruction, guidance and monitoring during the first days of IL-2 and histamine injections before self-administration at home. Only a few subjects required home care nursing for the injections.

### Evaluation of patients

Toxicity evaluation, physical examination and laboratory tests were performed every 5 weeks. Patients were evaluated for response after two and four cycles, if appropriate, and thereafter every third month until progressive disease (PD) was observed. Responses were reconfirmed after at least 4 weeks.

Objective response was defined according to the standard WHO criteria (Miller *et al*, 1981): (a) complete response (CR), defined as total disappearance of all clinical disease; (b) partial response (PR), defined as a reduction of more than 50% in the bidimensionally product diameter; (c) stable disease (SD), defined as a reduction of less than 50% or an increase in size of less than 25%; and (d) PD, defined as an increase in size of more than 25% in the bidimensionally product diameter or appearance of new lesions.

### Dose modifications

Toxicity was graded according to NCI common toxicity criteria, version 2.0. In general, no dose reduction of either study drug was done in case of grade 1 or 2 toxicity. In case of grade 3 or 4 toxicity, treatment was interrupted until toxicity returned to grade 1 or less or grade 2 or less. In most cases, IL-2 was then restarted at the 50% dose level and histamine at the 100% dose level.

### Management of toxicity

All patients received the following as needed to minimise toxicity: paracetamol, metoclopramide, ondansetron, loperamide, omeprazole, furosemide or mucopolysaccharidepolysulphate.

### Follow-up

Responses were evaluated every 3 months until PD. Patients were followed for survival every 3 months until death. No patients were lost to follow-up.

### Statistical methods

Treatment differences in response rates and other proportions were compared using Fisher's exact test. Time to PD was measured from first day of treatment until disease progression. Overall survival was measured from first day of treatment until death or last follow-up evaluation. The cumulated survival rate was estimated by the Kaplan–Meier method. The log-rank test was used to analyse survival differences among subgroups of patients. All calculations were performed using SPSS 11.0 statistical software. The survival data were updated 14 April 2005.

## RESULTS

Between June 1999 and August 2001, 41 patients were enrolled at the Department of Medical Oncology, Christie Hospital, Manchester, UK. Between August 2000 and August 2002, 63 patients were enrolled at the Department of Oncology, Aarhus, Denmark. The median follow-up time was 55 and 43 months, respectively, and the minimum length of follow-up 49 and 32 months, respectively. Separate analyses were carried out for the two studies as the trials were run independently. All patients met the eligibility criteria and were evaluable for toxicity. Number of patients receiving the planned drug dose was not significantly different between treatment arms in either of the two centres. Two patients in Manchester and four patients in Aarhus were nonevaluable for response as they stopped treatment within the first treatment cycle due to toxicity without signs of PD.

Table 1 lists baseline patient characteristics for the two patient populations. Patient characteristics were generally well balanced across treatment arms in the two studies, although slightly more Danish patients on the IL-2 arm had liver metastases (*P*=0.17) and slightly more Danish patients on the IL-2/HDC arm were males (*P*=0.10) (Table 1). When comparing the two study centres, it was noted that the UK patients more often had lung metastasis only (13/41 *vs* 2/63) than the Danish patients. More Danish patients had their primary kidney tumour *in situ* (26/63 *vs* 7/41), had higher frequency of lymph node metastases (38/63 *vs* 14/41), bone metastases (22/63 *vs* 4/41) and number of disease sites (three or more) (41/63 *vs* 10/41) than the UK patients.

### Tumour response

Based on an intention-to-treat analysis, overall response rates were not statistically significantly different between treatment groups (Table 2). However, for the Danish patients, a higher percentage of clinical benefit (CR+PR+SD) was noted in the IL-2/HDC group compared with the IL-2-alone group (58 *vs* 37%, respectively). This difference was of borderline significance (*P*=0.10). Response rates were identical in the two treatment groups in the UK study (Table 2).

### Survival

**Figure 1:**
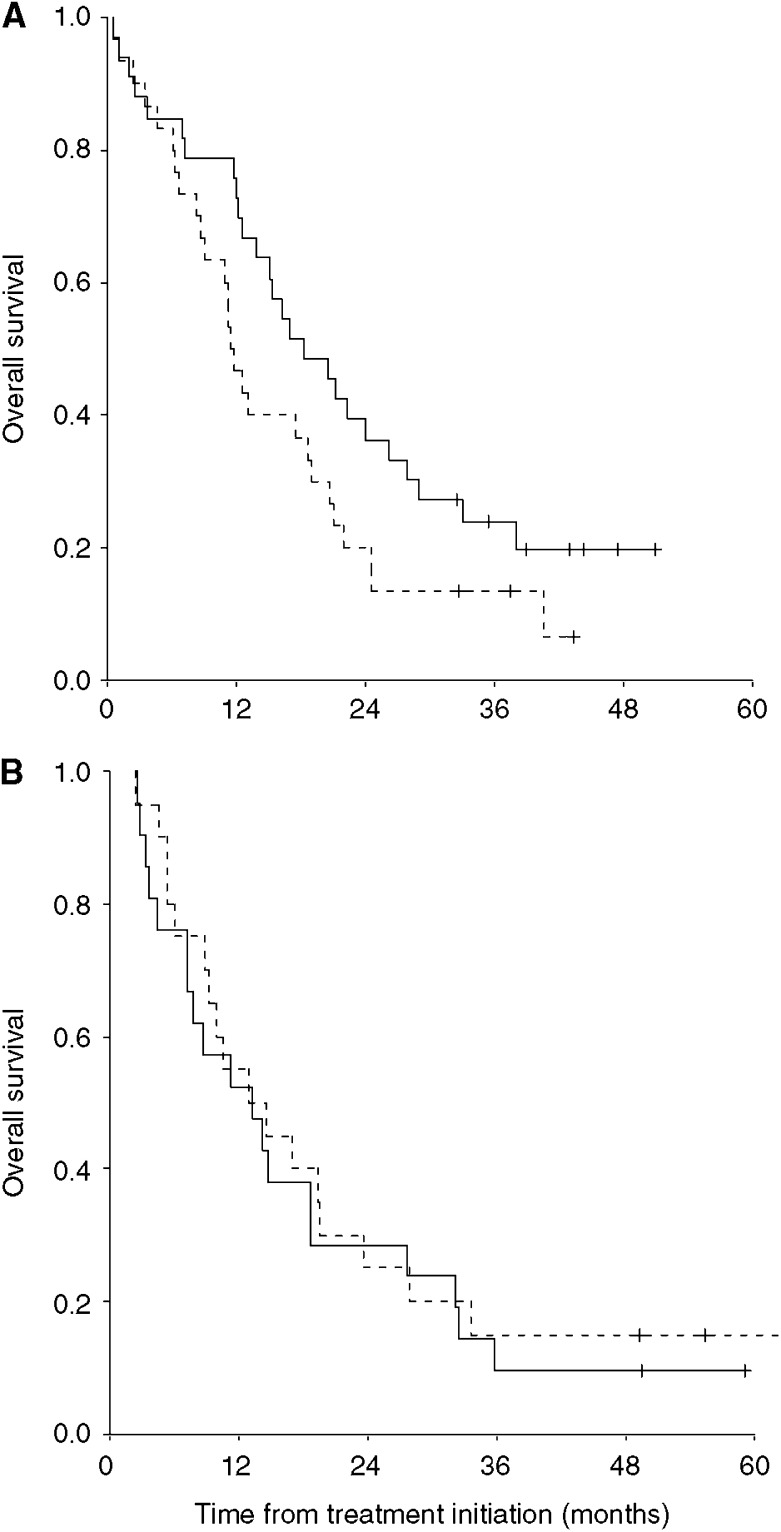
Kaplan–Meier estimates for overall survival of IL-2 plus histamine (—) *vs* IL-2 alone (- - -). Tick marks represent last date of follow-up. (**A**) Aarhus, *n*=63, (**B**) Manchester, *n*=41.

Kaplan–Meier survival distribution curves for the Danish patients (*n*=63) demonstrated a trend for improved survival for the IL-2 plus HDC group compared with the IL-2-alone group (Figure 1). Thus, median survival was improved from 11.4 months (IL-2) to 18.3 months (IL-2/HDC). This difference was of borderline significance (log rank *P*=0.07) (hazard ratio 0.61, 95% CI: 0.36–1.05) (Figure 1). The 1-year survival rate was statistically significantly improved from 47% for the IL-2-alone group to 76% for the IL-2 plus HDC group (*P*=0.03). However, Kaplan–Meier survival curves for the UK patients (*n*=41) were almost identical with no differences in median survival (IL-2, 12.9 months; IL-2/HDC, 13.2 months, *P*=0.6) or in the 1-year survival rate (IL-2, 55%; IL-2/HDC, 52%) (Figure 1).

At the time of analysis, 36 and 53 patients in the UK and the Danish study, respectively, had died. Thus, the event rate was 88 and 84%, respectively.

### Time to PD

**Figure 2:**
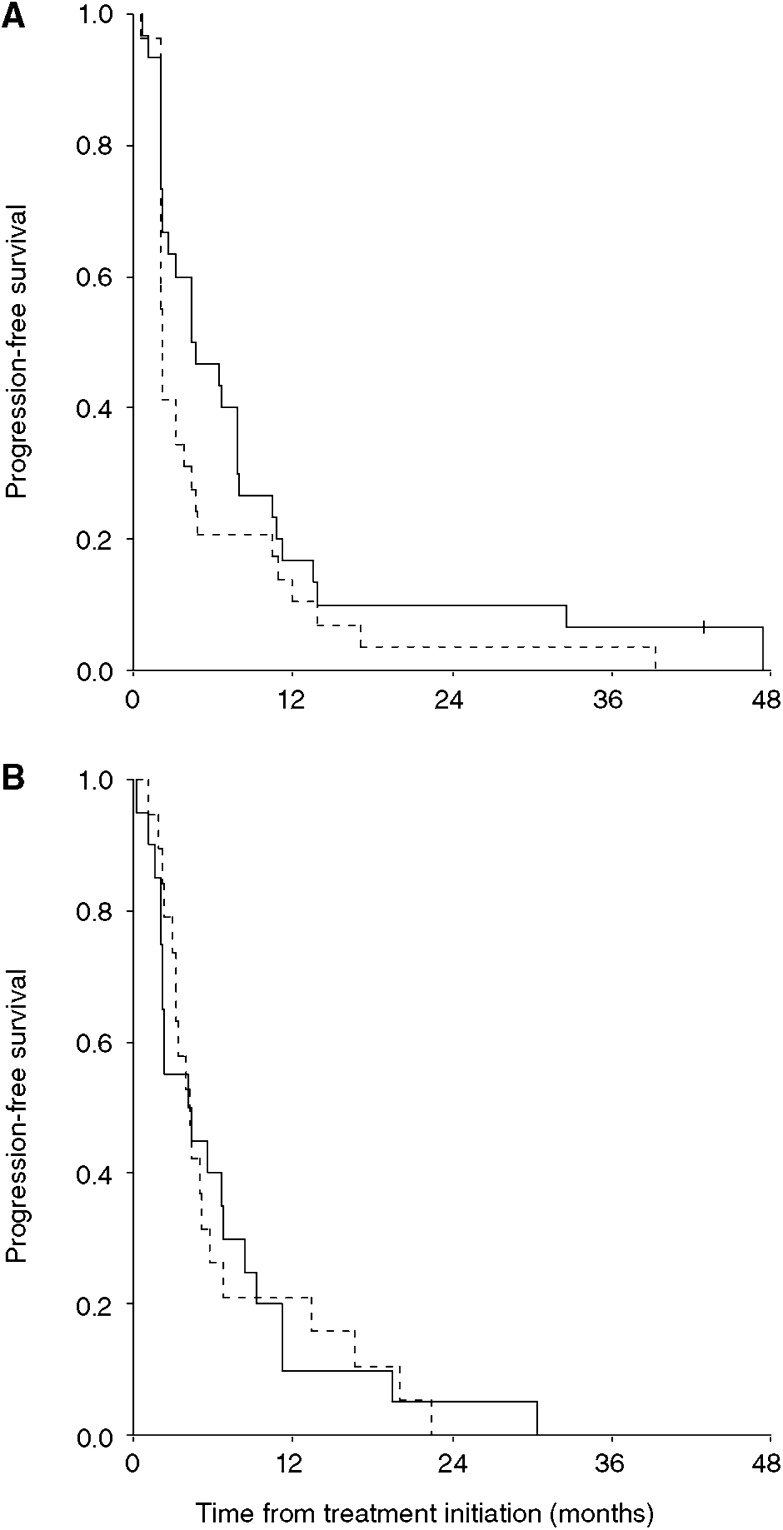
Kaplan–Meier estimates for progression-free survival of IL-2 plus histamine (—) *vs* IL-2 alone (- - -). (**A**) Aarhus, *n*=63, (**B**) Manchester, *n*=41.

Time to PD was not statistically significantly different for the two treatment groups in either of the two studies (Figure 2). In the Danish study, median time to PD was 2.2 months in the IL-2-alone group and median 4.5 months in the IL-2 plus HDC group (*P*=0.13), whereas time to PD was identical in the two treatment groups in the UK study (Figure 2). At the time of analysis, only one patient had not progressed, giving a censoring rate of 1% in total for the two studies.

### Toxicity

At least 70% of patients received two cycles of treatment and at least 23% of patients received four cycles of treatment in the two studies. A total of 4500 histamine injections were given as self-administration at home without medical supervision. In general, toxic effects were minor to moderate. All patients had at least one toxic side effect. Table 3 lists grade 3/4 toxicity to treatment. There was generally more reported toxicity among the Danish patients compared with the UK patients. Concerning nausea and vomiting, the toxicity was more pronounced in the UK study. However, toxicity was not significantly different for IL-2-alone compared with IL-2 plus HDC in either of the studies. In total, only three of the 104 patients had grade 4 toxicity (lethargy, *Escherichia coli* sepsis and thrombocytopenia/bowel infarction, respectively). These three patients received IL-2/HDC and two of the episodes were fatal. One patient died due to septicaemia, which was considered unrelated to the study drug medication. The other patient died due to thrombocytopenia/bowel infarction and this death was possibly related to treatment.

## DISCUSSION

Since its introduction to the clinic in 1985, IL-2 remains the only established cytokine approved by the US Food and Drug Administration for the treatment of mRCC. Despite 20 years of investigations, no combination therapy has proved better than IL-2 treatment alone in terms of long-term survival (Negrier *et al*, 1998; McDermott *et al*, 2005).

Histamine dihydrochloride as an adjunct to IL-2 is an example of translational research from basic biology to clinical trials. A large number of *in vitro* and *in vivo* observations from independent laboratories have supported the observation of oxidative suppression of NK and T cells by phagocytes (i.e. monocytes, macrophages and neutrophils) (Seaman *et al*, 1982; Finke *et al*, 1993; Tartour *et al*, 1995; Hansson *et al*, 1996a; Kono *et al*, 1996; Saio *et al*, 2001). Hellstrand and co-workers have demonstrated that histamine protects NK and T cells against oxygen radical-induced dysfunction and apoptosis by specifically blocking the formation and release of hydrogen peroxide (H_2_O_2_) from phagocytes, and moreover, maintains the activation of NK and T cells by IL-2 (Hellstrand and Hermodsson, 1986, 1990, 1991; Hellstrand *et al*, 1990, 1994a, 1994b; Asea *et al*, 1996; Hansson *et al*, 1996b, 1999). This potential mechanism has been explored clinically in acute myelogenous leukaemia (Brune and Hellstrand, 1996), chronic hepatitis C (Lurie *et al*, 2002), multiple myeloma (Mellqvist *et al*, 1999), metastatic melanoma (Agarwala *et al*, 2002, 2004; Schmidt *et al*, 2002) and mRCC (Donskov *et al*, 2002).

We introduced histamine in mRCC in combination with low-dose IL-2 and IFN-*α* (Donskov *et al*, 2002). In parallel with the clinical trial, we obtained serial blood samples and tumour biopsies, searching for a potential histamine effect *in situ* (Donskov *et al*, 2004). However, in that low-dose schedule of IL-2 and IFN-*α*, histamine did not appear to add efficacy with respect to response (Donskov *et al*, 2002). Moreover, no discernable differences in the examined immunologic parameters in blood and tumour tissue could be detected between histamine- and nonhistamine-treated patients (Donskov *et al*, 2004).

However, the question of whether histamine might improve efficacy with higher doses of IL-2 formed the basis for the present studies representing the first *randomised* trials in mRCC evaluating the efficacy and safety of subcutaneous IL-2 in combination with HDC. Thus, in the present study, we have doubled the dose of IL-2 compared to that in our first IL-2/IFN/histamine-study and the applied IL-2 dose can be considered to be an intermediate dose level. Protocol criteria were similar in the two studies. However, the Danish patients had a significantly greater disease burden than UK patients. The outcome of the individual studies differed as the Danish study showed a statistically significant 1-year survival benefit and a trend towards benefit in both overall survival and clinical benefit (CR+PR+SD) in favour of IL-2/HDC, whereas the UK study was negative for all end points. This means that the potential effect of histamine was only indicated in the Danish study and by that in the group of patients with the worse prognosis. The explanation for this difference in the outcome of the two trials is unclear, but it should be emphasised that the different outcome may have been obtained by pure chance.

In the Danish patients, we also assessed the oxidative stress hypothesis by monitoring cells of potential benefit (i.e. NK and T cells) and cells of potential harm (i.e. monocytes/macrophages and neutrophils) simultaneously in blood and tumour tissue, before and during treatment. These assays provide compelling evidence for circulating monocytes and neutrophils as powerful negative prognostic factors for IL-2 based immunotherapy and establish a biological rationale for the addition of histamine to IL-2 in mRCC. Thus, targeting H_2_O_2_ by histamine seems to enhance the antitumour activity of IL-2 *in situ* in a subgroup of patients with low monocytes/neutrophils or high NK cells. These results will be published separately.

Toxicity was in general manageable in an outpatient setting, although we observed one possible toxic death. Adverse events noted in patients treated with the combination of IL-2 and histamine were almost similar in type and frequency as toxicities in patients treated with IL-2 alone.

In conclusion, the outcome of the individual studies differed as the Danish study showed a trend towards benefit in favour of IL-2/HDC, whereas the UK study was negative for all end points. A randomised phase III trial is warranted to clarify the potential role of adding histamine to IL-2 in mRCC.

## Figures and Tables

**Table 1:** Baseline patient characteristics

	**Aarhus, Denmark**		**Manchester, United Kingdom**	
	**IL-2/HDC (*N*=33) *n***	**IL-2 (*N*=30) *n***	**IL-2/HDC (*N*=21) *n***	**IL-2 (*N*=20) *n***
Patients evaluable for response	30	29	20	19
Median age, years (range)	56 (31–69)	60 (36–69)	57 (37–70)	53 (28–71)
Male	26	17	16	18
				
*Karnofsky performance status*				
100	19	12	3	4
90	10	13	11	12
80	2	2	6	3
70	2	3	1	1
				
Metastasis-free interval ⩽1 year	23	21	18	14
				
*Prior therapy*				
Nephrectomy	19	19	16	18
Embolisation	—	—	2	—
Excision of metastatic lesions	4	2	1	2
Radiotherapy	2	1	2	—
Thalidomide/hormones	—	1	—	1
Immunotherapy	—	—	—	3
				
*Number of disease organ sites*				
1	3	3	8	7
2	8	8	6	10
3 or more	22	19	7	3
				
*Most common sites of disease*				
Primary kidney tumour	14	12	5	2
Local recurrence kidney bed	4	6	5	4
Lung/pleura	26	23	17	16
Lung only	2	0	7	6
Lymph node	21	17	7	7
Liver	3	7	2	2
Bone	10	12	3	1
				
*MSKCC prognostic criteria* a				
Favourable prognosis	8	5	4	7
Intermediate prognosis	21	19	11	7
Poor prognosis	4	6	1	1
Missing values	—	—	5	5

a Memorial Sloan-Kettering Cancer Center. *J Clin Oncol* 1999; **17**: 2530–2540.

**Table 2:** Response to treatment

	**Aarhus, Denmark**			**Manchester, United Kingdom**		
	**IL-2/HDC (*N*=33)**	**IL-2 (*N*=30)**		**IL-2/HDC (*N*=21)**	**IL-2 (*N*=20)**	
	***n*** **(%)**	***n*** **(%)**	***P***	***n*** **(%)**	***n*** **(%)**	***P***
Evaluable for response	30	29		20	19	
CR	2	1		0	0	
PR	2	0		3	3	
SD	15	10		9	8	
PD	11	18		8	8	
Overall response (ITT)	4 (12)	1 (3)	0.36	3 (14)	3 (15)	1.0
Clinical benefit (ITT)	19 (58)	11 (37)	0.10	12 (57)	11 (55)	1.0

CR=complete response; PR=partial response; SD=stable disease; PD=progressive disease; clinical benefit=CR+PR+SD; ITT=intention-to-treat analysis.

**Table 3:** Grade 3 or 4 toxicity to treatment

	**Aarhus, Denmark**		**Manchester, United Kingdom**	
	**IL-2/HDC (*N*=33)**	**IL-2 (*N*=30)**	**IL-2/HDC (*N*=21)**	**IL-2 (*N*=20)**
	**%**	**%**	**%**	**%**
Nausea			14	
Vomiting			5	5
Diarrhoea		3		
Stomatitis				5
Weight loss/anorexia		3		
Dyspnea	6	13	5	
Cough		7		
Bronchospasm		3		5
Dehydration		3		
Lethargy	18	20	10	10
Local injection site reaction	9	3		
Urticaria/facial swelling			5	5
Flu-like symptoms		7	10	5
Confusion/memory loss	6	7		
Hypotension	3			
Headache			5	
Oedema			5	
Tremor	3			
Pain		3		
Creatinin rise		3		
Hyperkalemia	3	3		
Others	6			
At least one SAE	49	43	19	35

Worst grade 3/4 related to treatment according to NCI common toxicity criteria version 2.0. SAE=severe adverse event.
